# ﻿Novelties on the genus *Vaccinium* (Ericaceae) from Hainan, China: a new species and a new record for the country

**DOI:** 10.3897/phytokeys.202.82786

**Published:** 2022-07-29

**Authors:** Xue-He Ye, Qing-Long Wang, Ming-Zhong Huang, Yi-Hua Tong

**Affiliations:** 1 Key Laboratory of Plant Resources Conservation and Sustainable Utilization & Guangdong Provincial Key Laboratory of Applied Botany, South China Botanical Garden, Chinese Academy of Sciences, Guangzhou, 510650, China South China Botanical Garden, Chinese Academy of Sciences Guangzhou China; 2 Center of Conservation Biology, Core Botanical Gardens, Chinese Academy of Sciences, Guangzhou, 510650, China Core Botanical Gardens, Chinese Academy of Sciences Guangzhou China; 3 Zhongkai University of Agriculture and Engineering, Guangzhou, 510225, China Zhongkai University of Agriculture and Engineering Guangzhou China; 4 Tropical Crops Genetic Resources Institute, Chinese Academy of Tropical Agricultural Sciences, Haikou, 571101, China Tropical Crops Genetic Resources Institute Haikou China

**Keywords:** Morphology, new species, taxonomy, *Vacciniumpubicalyx*

## Abstract

Here we describe a new species, *Vacciniumpseudopubicalyx*, and report a new record for the flora of China, *V.viscifolium*, both from Hainan Province. *Vacciniumviscifolium* also represents the first record of V.sect.Euepigynium for China. Detailed descriptions and illustrations with analytical photographs of the two taxa are provided.

## ﻿Introduction

The genus *Vaccinium* L., with about 450–500 species distributed worldwide, is the largest genus of the tribe Vaccinieae in Ericaceae (Fang 1991; Fang and Stevens 2005; Vander Kloet and Dickinson 2009; POWO 2022). In China, 97 species of *Vaccinium* have been recorded, with several new species or records reported from the country after the revision completed by Fang and Stevens (2005) in “Flora of China”, such as *V.eberhardtii* Dop, *V.damingshanense* Y. H. Tong & N. H. Xia, *V.napoense* Y. H. Tong & N. H. Xia, *V.zhangzhouense* Y. H. Tong, Y. Y. Zhu & N. H. Xia, and *V.motuoense* Y. H. Tong & Y. J. Guo (Fang 1991; Fang and Stevens 2005; Tong and Xia 2015; Tong et al. 2018; Tong et al. 2020; Tong et al. 2021a, b). Three species of *Vaccinium* are recorded from Hainan Province in “Flora of Hainan”, viz. *V.bracteatum* Thunb., *V.chunii* Merr. ex Sleumer and *V.hainanense* Sleumer, among which *V.hainanense* is endemic to Hainan (Instituti Botanici Austro-Sinensis Academiae Sinicae 1974).

During a recent field trip to Hainan, two species never recorded from the province were respectively found in Wuzhi Mountain and Mazui Mountain. The one from Wuzhi Mountain is similar to *V.pubicalyx* Franch. and V.bracteatumThunb.var.chinense (Lodd.) Chun ex Sleumer due to their similar habit and leaf blade shape. The other one from Mazui Mountain firstly reminded us of *V.hainanense*, a species that has never been collected again since 1933 when the type specimens were collected, and owns the similar leathery leaf blades with an obtuse or abruptly obtuse-acute apex as this unknown species. However, its pinnipalmate leaf venation and glabrous floral disk are rather different from *V.hainanense*.

After a detailed examination of our materials and possible similar species from China and neighbouring countries (King and Gamble 1910; Dop 1930; Sleumer 1967; Pham 1999; Nguyen 2005; Newman et al. 2007; Watthana 2015), we concluded that the species from Wuzhi Mountain is new to science, and the other from Mazui Mountain is *V.viscifolium* King & Gamble, which represents a new record for the flora of China. Thus, we report these novelties as follows.

## ﻿Taxonomy

### ﻿ Vaccinium pseudopubicalyx

Taxon classificationPlantaeEricalesEricaceae

X. H. Ye, Q. L. Wang & Y. H. Tong sp. nov.

6F2374C0-1F36-5C20-A9AF-1A858373C8CC

urn:lsid:ipni.org:names:77302514-1

Figs 1 , 2 , 3

#### Type.

China, Hainan, Wuzhishan City, Wuzhi Mountain, montane summit scrub, 18.89°N, 109.69°E,1867 m a.s.l., 20 March 2020, *Yi-Hua Tong*, *Xue-He Ye*, *Xin-Ting Ma & Qing-Long Wang YXH-18* (holotype: IBSC; isotypes: IBSC, ATCH).

**Figure 1.**
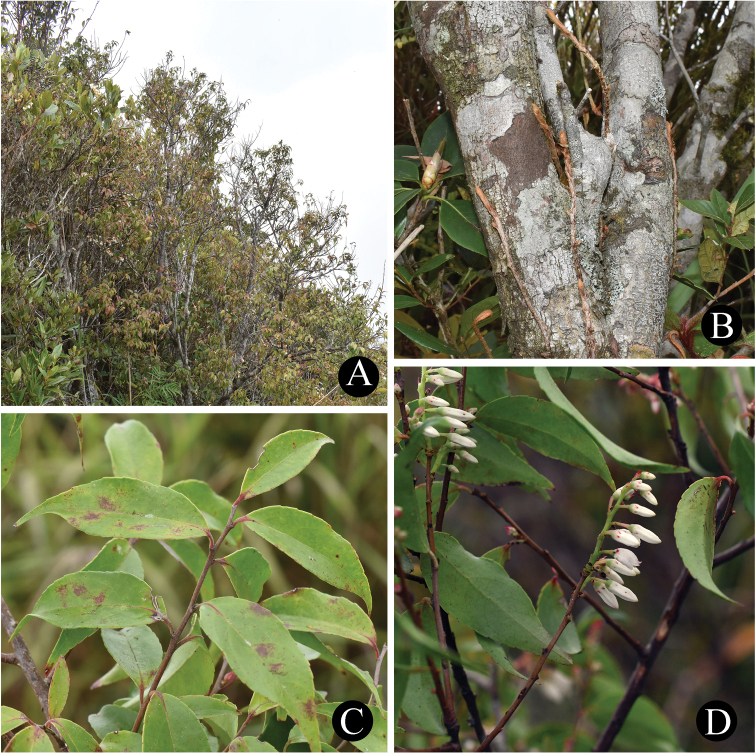
*Vacciniumpseudopubicalyx***A** habit **B** trunk **C** leafy branch **D** flowering branch. Photos **A–C** by Y. H. Tong; **D** by X. H. Ye.

#### Diagnosis.

**Figure 2.**
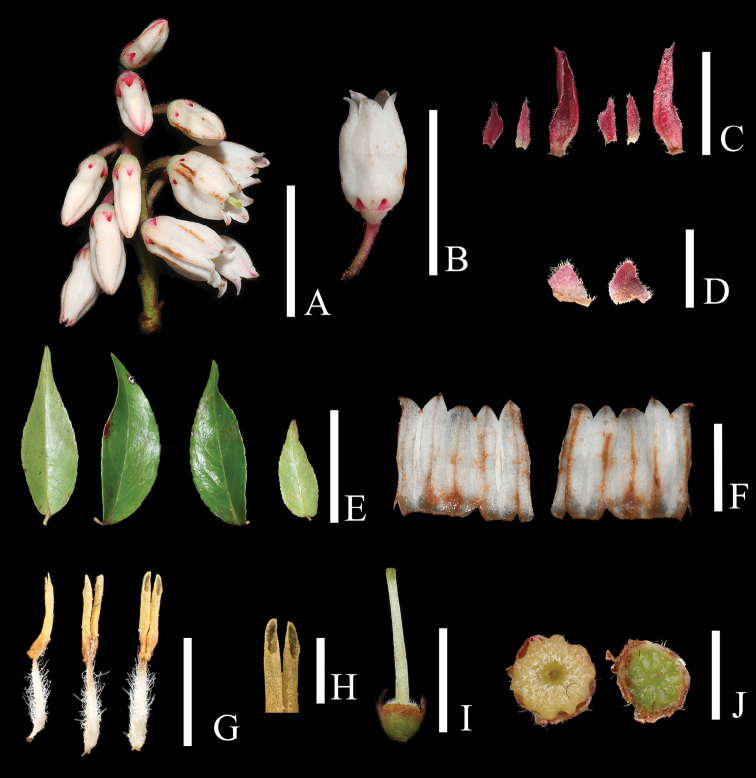
*Vacciniumpseudopubicalyx***A** inflorescence **B** flower **C** two bracteoles and one floral bract, adaxial (left) and abaxial (right) view **D** calyx lobe, abaxial (left) and adaxial (right) view **E** leaves **F** opened corolla, adaxial (left) and abaxial (right) view **G** stamens, lateral (left), abaxial (middle) and adaxial (right) view **H** anther pores **I** hypanthium, disk and style with front two calyx lobes removed **J** ovary, apical (left) and transverse (right) view. Scale bars: 3 cm (**E**); 1 cm (**A, B**); 5 mm (**F, I**); 3 mm (**D, G**); 2 mm (**D, J**); 1 mm (**H**). Photos by X. H. Ye.

*Vacciniumpseudopubicalyx* is morphologically similar to *V.pubicalyx* and its varieties, but can be distinguished by having fewer pairs of secondary veins (3–5 (Fig. 2E) vs. 5–9 (Fig. 5A, B)), smaller (2–3.5 × ca. 1 mm vs. 5–8 × ca. 1.5–2 mm) and lanceolate (vs. ovate) floral bracts, young twigs and inflorescences rachis white-pubescent (vs. brownish-tomentose), and anthers with smaller dorsal spurs (0.5–1 mm vs. ca 4 mm, Fig. 5F).

#### Description.

Evergreen terrestrial shrubs or small trees, 2–4 m tall; much branched, young twigs pubescent, glabrescent. Petioles 3–5 mm long, pubescent; blades elliptic or ovate, 2.2–5 × 0.8–1.9 cm, chartaceous, glabrous on both sides, midvein prominent on both sides, pubescent, glabrescent abaxially, secondary veins 3–5 pairs, visible in vivo, inconspicuous adaxially and slightly raised abaxially when dry, base broadly cuneate, margin serrulate, apex acuminate, shortly caudate. Perennating buds monomorphic. Inflorescence pseudo-terminal or axillary on distal part of twigs, racemose, with 10–13 flowers, rachis 1.4–2.5 cm long, densely pubescent; floral bract caducous, 1, inserted at the base of pedicel, lanceolate to linear, 2–3.5 × ca. 1 mm, glabrous adaxially, pubescent abaxially, margin entire, ciliate; bracteoles 2, unequally inserted at the lower part of pedicel, long ovate-triangular to linear, 1–1.6× ca. 1 mm, glabrous adaxially, pubescent abaxially, margin entire, ciliate; pedicel articulated between pedicel and calyx, 3–4.5 mm long, densely pubescent. Hypanthium cupuliform, 1–2 × 1.5–2 mm, pubescent; calyx lobes triangular to broadly triangular, 1–2.5 × 1–1.5 mm, glabrous adaxially, pubescent abaxially, margin entire, ciliate. Corolla white, tinged with red, tubular, sometimes slightly urceolate, 4.5–8 × 3.5–4.5 mm, pubescent outside, pilose inside; corolla lobes slightly reflexed, small, triangular, 1–1.8 × 1–1.5 mm; stamens 10, dimorphic, 4.5–5 mm long, filaments 2–3 mm long, densely pilose, anthers dorsifixed with 2 minute spurs, ca. 2.5 mm long, thecae ca. 1 mm long, papillate, spurs on antesepalous anthers ca. 0.1 mm long, those on antepetalous anthers ca. 0.05 mm long, inconspicuous, tubules 1.5–2 mm long, 1.5–2 times as long as anther thecae, apex poricidal, pores 0.45–0.6 mm long, teeth absent; ovary inferior, pseudo-10-locular, disk disciform, 10-ridged, pilose, style cylindrical, 6–6.5 mm long, glabrous, stigma punctate. Fruits not seen.

**Figure 3.**
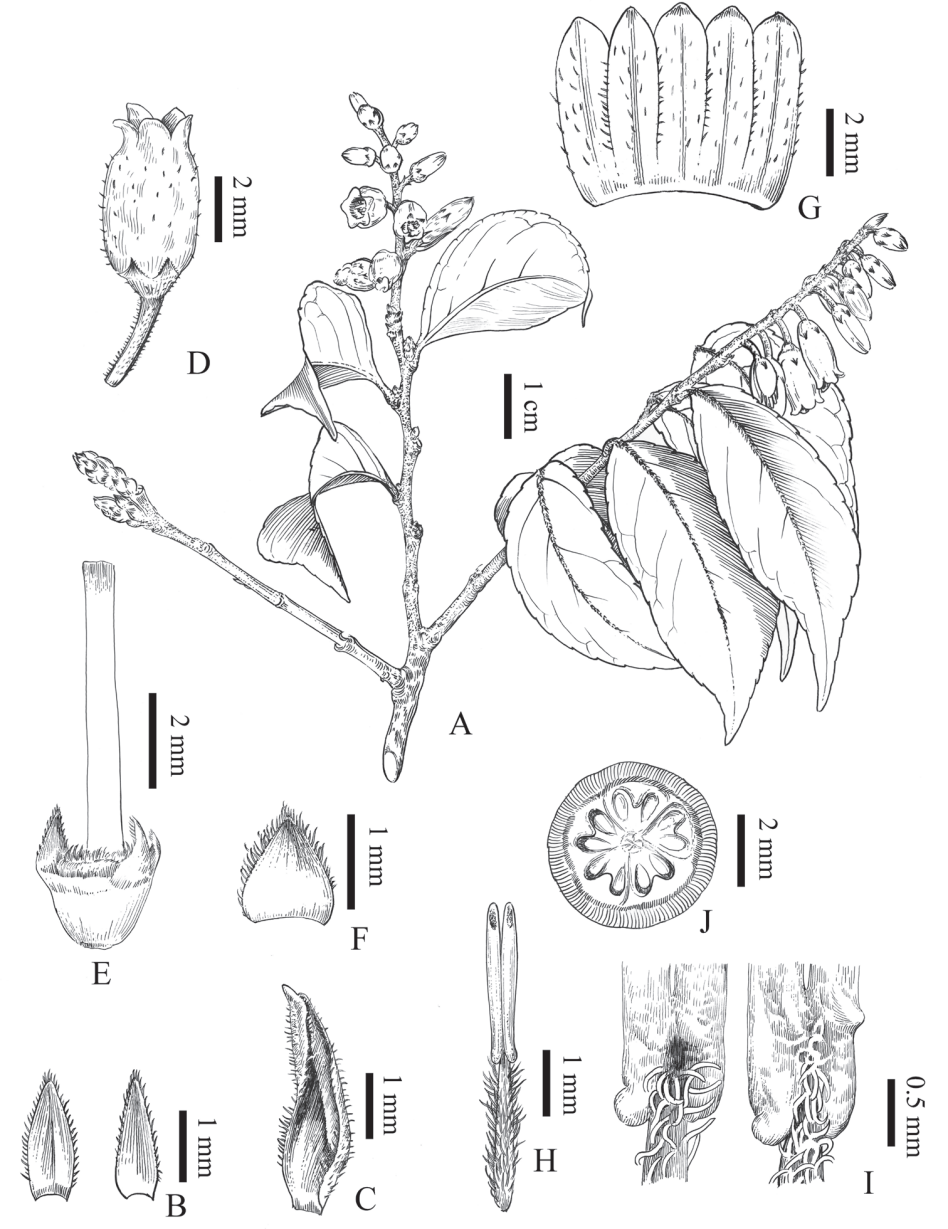
*Vacciniumpseudopubicalyx***A** flowering branches **B** bracteoles, adaxial (left) and abaxial (right) view **C** floral bract, adaxial view **D** flower **E** hypanthium, disk and style with front one calyx lobe removed **F** calyx lobe, adaxial view **G** opened corolla, adaxial view **H** stamens, adaxial view **I** dorsal anther spurs of antepetalous anthers (left) and antesepalous anthers (right) **J** ovary, transverse view. Drawn by Mr. D. H. Cui.

#### Etymology.

The specific epithet *pseudopubicalyx*, a noun in apposition, alludes to close alliance to *Vacciniumpubicalyx*.

#### Vernacular name.

拟毛萼越橘 (Chinese pinyin: nǐ máo è yuè jú).

#### Phenology.

Flowering in January–June; fruiting time unknown.

#### Distribution and habitat.

**Figure 4.**
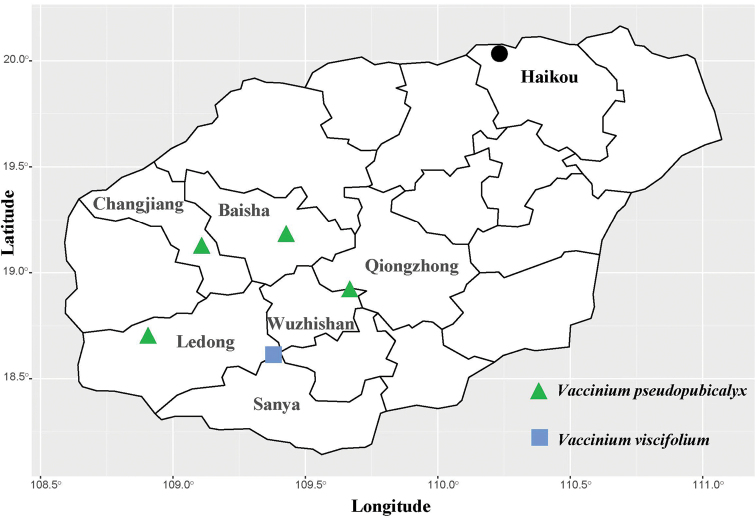
The distribution of *Vacciniumpseudopubicalyx* and *V.viscifolium* in Hainan Island (China).

*Vacciniumpseudopubicalyx* has been found at four localities in Hainan, viz. the type locality, Bawangling National Nature Reserve (19.12°N, 109.08°E), Jianfengling Tropical Forest Nature Reserve (18.72°N, 108.91°E) and Yinggeling National Nature Reserve (19.18°N, 109.45°E) (Fig. 4). It grows in montane forests or scrub on the mountain summits at elevations from 1122 to 1867 m.

#### Taxonomic notes.

Besides the differences mentioned in the diagnosis, *Vacciniumpseudopubicalyx* and *V.pubicalyx* are strictly allopatric: the former one is endemic to Hainan, while the other one is distributed in southwest China and Myanmar.

The new species has also been occasionally identified as V.bracteatumvar.chinense (Champ. ex Benth.) Chun ex Sleumer (Zhang et al. 2007; Xing et al. 2012). It can be easily separated from V.bracteatumvar.chinense by its inconspicuous (vs. prominent) secondary veins on adaxial surface of leaf blade. Besides, the elliptic or ovate (vs. rhombic-elliptic or lanceolate-elliptic) leaf blades with fewer pairs of secondary veins (3–5 vs. 5–7), shorter inflorescence rachis (1.4–2.5 cm vs. 4–10 cm), smaller (2–3.5 × ca. 1 mm vs. 5–20 × 1–3 mm), entire (vs. serrated) and lanceolate to linear (vs. ovate to oblong-ovate) floral bracts, and different ratio of the length of anther tubules to anther thecae (1.5–2 vs. 2–2.5) can also separate the new species from V.bracteatumvar.chinense.

*Vacciniumpseudopubicalyx* should be assigned to sect. Eococcus Sleumer, because its morphological characteristics match well with those of that section, such as a terrestrial habit, serrate leaf blades, axillary racemes with elongate rachis, deciduous bracts, articulation between hypanthium and pedicel, spurred anthers and a pseudo-10-locular ovary. According to Sleumer (1941), the main difference between sect. Eococcus and sect. Bracteata Nakai is that the former has caducous bracts during anthesis, whereas the latter has persistent ones. However, when examining specimens of *V.iteophyllum* Hance, a widespread species (distributed in almost every province in southern China) placed in sect. Eococcus by Sleumer (1941), we observed that some populations (e.g., *Y. H. Tong & L. Bai 12062414*, IBSC) also have persistent bracts even when fruiting. Thus, the delimitation of the two sections appears to not be natural, and the relationship of the two sections needs further study.

#### Additional specimens examined.

*Vacciniumpseudopubicalyx* (paratypes): China. Hainan: Baisha County, Yuanmen Town, Yinggeling National Nature Reserve, main peak, 31 May 2005 (fl.), *F. W. Xing*, *Rong-Jing Zhang & Yu-Shi Ye 16371* (IBSC); ibid., same date as above, *Sai-Chit Ng 5766* (HK, not seen); Changjiang County, Bawangling National Nature Reserve, mountain top, 1380 m a.s.l., 6 January 1964, *Pei Zeng 13511* (HITBC); ibid., the second peak (Axe Peak), 27 April 1988 (fl.), *Ze-Xian Li & Fu-Wu Xing 4004* (IBSC0528562); ibid., the second peak (Axe Peak), June 1983 (fl.), *Guo-Ai Fu 3408* (IBSC0420642); Ledong County, Jianfengling Tropical Forest Nature Reserve, 1400 m a.s.l., 25 January 1984 (fl.), *Shi-Man Huang 306* (IBSC0420202); ibid., the second peak, 1220 m a.s.l., 29 November 1957, *Qi-Cai He 90777* (IBSC0420580).

*Vacciniumpubicalyx*: China. Yunnan: Tali [Dali Profecture], Houang-kia-pin [Huangjiaping], 4 July 1888, *P. J. M. Delavay 3311* (syntypes: P04484707, image; P04484708, image; P04484709, image; L008184, image); [Tengchong City], hills at the north end of the Tengyueh Valley, May 1912, *G. Forrest 7637* (IBSC0457011; K00780601, image; E00327780, image; E00327781, image).

**Figure 5.**
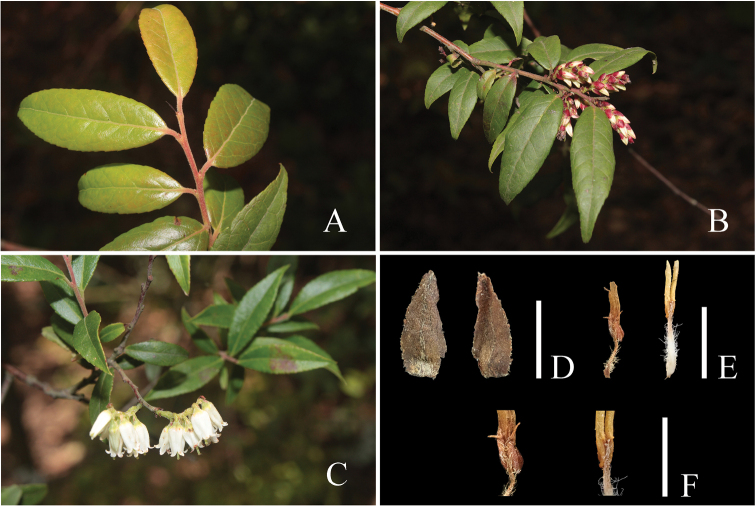
*Vacciniumpubicalyx* and comparison of stamen of *V.pubicalyx* and *V.pseudopubicalyx***A** young leafy branch of *V.pubicalyx***B** branch with young inflorescences of *V.pubicalyx***C** flowering branches of *V.pubicalyx***D** floral bract of *V.pubicalyx*, abaxial (left) and adaxial (right) view **E** stamens of *V.pubicalyx* (left) and *V.pseudopubicalyx* (right) **F** dorsal anther spurs of *V.pubicalyx* (left) and *V.pseudopubicalyx* (right). Scale bars: 3 mm (**D, E**); 2 mm (**F**). Materials of *V.pubicalyx* in **D–F** from *G. Forrest 7637* (IBSC0457011), and those of *V.pseudopubicalyx* in **E–F** from type. Photos **A, C** by H. B. Ding; **B** by Y. H. Tong; **D–F** by X. H. Ye.

Vacciniumbracteatumvar.chinense: China. Hong Kong: *Champion s. n.* (K00780589, image).

### ﻿ Vaccinium viscifolium

Taxon classificationPlantaeEricalesEricaceae

King & Gamble in J. Asiat. Soc. Bengal, Pt. 2, Nat. Hist. 74(1): 63. 1906.

42E147F2-0548-5845-A19B-2C3F0C86C98D

Figs 6 , 7

#### Type.

Malaysia, Perak, *Scortechini 405* (lectotype CAL, not seen, designated by Sleumer in 1961: 84; isolectotypes BM000996430, image!, K, not seen, P00647874, image!)

#### Description.

Evergreen terrestrial shrubs or trees, up to 6 m tall; young twigs pubescent, glabrescent. Petioles flat, 0.5–1.2 cm long, pubescent; blades elliptic, oblanceolate, obovate or oblong-obovate, 4.8–11.1 × 1.3–3.4 cm, coriaceous, stipitate-glandular on both sides, pinnipalmate, midvein prominent abaxially, flat or slightly sunken adaxially, secondary veins 3–6 pairs, in vivo visible adaxially, flat or slightly raised, inconspicuous abaxially, in sicco inconspicuous on both sides, apex obtuse or rounded, margin entire, slightly revolute, with 1–3 pairs of glands at the basal part, base attenuate, extended. Perennating buds monomorphic. Inflorescence axillary, racemose, with 11–18 flowers; rachis 3.8–5 cm long, sparsely stipitate-glandular; bract caducous, 1, inserted at the base of pedicel, ovate or lanceolate, 9–16 × 3–6.5 mm, glabrous, margin entire, involute; bracteoles oppositely inserted at the base of pedicel, 2, linear, 1.5–2.5× ca. 0.5 mm, glabrous on both sides, margin entire, ciliate; pedicel articulated between pedicel and calyx, 3–5.5 mm long, glabrous. Hypanthium cupuliform, 2–2.5 × 2.5–3 mm, glabrous; lobes triangular to broadly triangular, 1–2.5 × 1.5–2 mm, glabrous on both sides, margin ciliate. Corolla pinkish to white, cylindric-urceolate, 8–10 × 4–5 mm, glabrous on both sides; lobes reflexed or slightly spreading, small, ovate or triangular, 1.5–2 × 1.5–2 mm, glabrous abaxially, papillate adaxially; stamens 10, dimorphic, 6–7 mm long, filaments swollen at base, 3–3.5 mm long, densely pilose, anthers without dorsal spurs, 3–3.5 mm long, thecae 1–1.5 mm long, papillate, tubules 1.5–2.5 mm long, 1.5–2 times as long as anther thecae, apex poricidal, pores ca. 0.5 mm long, antesepalous anthers with 1 tooth at apex of anther tubules, antepetalous anthers with 2 teeth at apex of anther tubules, teeth ca. 0.5 mm long; ovary inferior, pseudo-10-locular, disk disciform, 10 ridged, glabrous, style cylindrical, 0.8–0.9 cm long, glabrous, stigma truncate. Young fruits glaucous, subglobose, persistent calyx lobes incurved, nearly covering disk.

**Figure 6.**
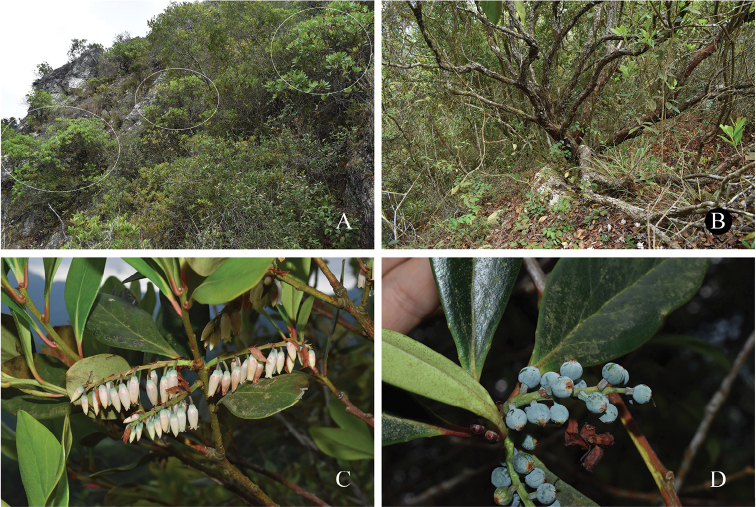
*Vacciniumviscifolium***A** habitat **B** habit **C** flowering branch **D** fruiting branch. Photos by Y. H. Tong.

#### Vernacular name.

槲寄生叶越橘 (Chinese pinyin: hú jì shēng yè yuè jú).

#### Distribution and habitat.

*Vacciniumviscifolium* is distributed in China (Hainan, Fig. 4), Southern Indochina to Peninsular Malaysia. So far, Hainan is the northernmost distribution locality of this species. It grows on open limestone of mountainsides or mountain ridges in montane forests at an elevation of ca. 750 m in Hainan.

#### Taxonomic notes.

*Vacciniumviscifolium* has two varieties, viz. the nominate variety and V.viscifoliumvar.bicalcaratum Sleumer. The latter differs from the former by having leaves with a shortly (ca. 1 cm) and obtusely attenuate (vs. obtuse or rounded) apex and anthers with (vs. without) 2 distinct dorsal spurs (Sleumer 1967). The plants from Hainan should belong to the nominate variety, as they have leaves with an obtuse or rounded apex and anthers without dorsal spurs.

*Vacciniumviscifolium* was initially assigned to sect. Euepigynium Schltr. by Sleumer (1941). However, when compiling *Flora Malesiana*, he reassigned it to sect. Bracteata, which was recognized with an extremely broad sense to include 163 species consisting of members from several sections, such as sect. Bracteata s.s., sect. Nesococcus H. F. Copel., and sect. Euepigynium (Sleumer, 1967). Stevens (1969) pointed out that the leaves of the species of sect. Euepigynium with an entire margin and plinerved venation were very different from those of sect. Bracteata, which have a serrate margin and pinnate venation. Vander Kloet and Dickinson (2009) also treated sect. Euepigynium and sect. Bracteata as two distinct sections. Moreover, the marginal or basal glands often occur to sect. Euepigynium, and are absent in the species of sect. Bracteata s.s. without exception. Thus, we do not adopt Sleumer’s broad sense of sect. Bracteata (Sleumer, 1967), and prefer to agree with the assignment of *V.viscifolium* to sect. EuepigyniumSleumer (1941). Whereas sect. Euepigynium is only found in Malesiana and Indochina formerly, *Vacciniumviscifolium* is the first species of sect. Euepigynium reported from China.

**Figure 7.**
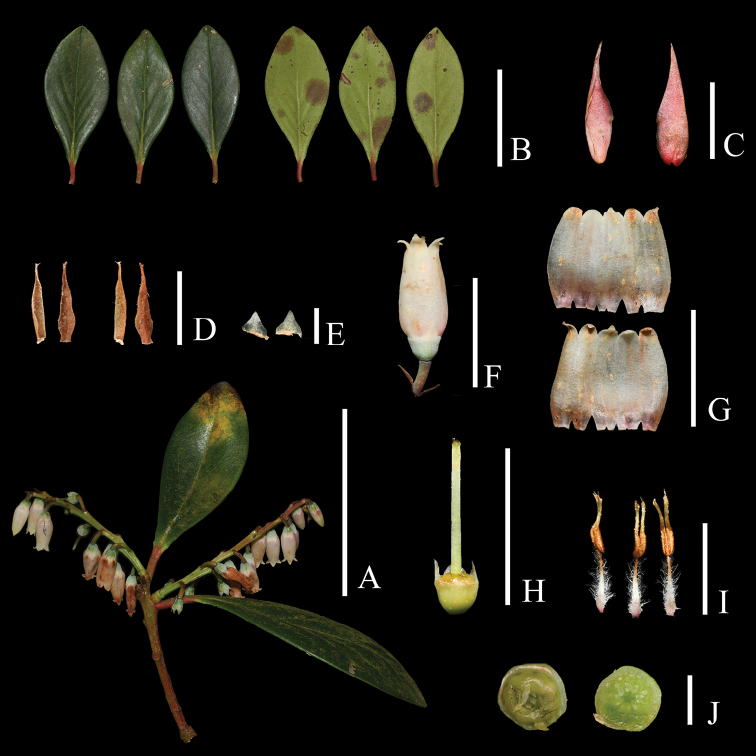
*Vacciniumviscifolium***A** flowering branch **B** leaves **C** floral bract, adaxial (left) and abaxial (right) view **D** bracteoles, adaxial (left) and abaxial (right) view **E** calyx lobe, adaxial (left) and abaxial (right) view **F** flower **G** opened corolla, adaxial (up) and abaxial (below) view **H** hypanthium, disk and style with front two calyx lobes removed **I** stamens, lateral (left), abaxial (middle) and adaxial (right) view **J** ovary, apical (left) and transverse (right) view. Scale bars: 5 cm (**A, B**); 1 cm (**C, F, G, H**); 5 mm (**I**); 2 mm (**D, E, J**). Photos by X. H. Ye.

#### Additional specimens examined.

*Vacciniumviscifolium*: China. Hainan: Ledong County, the ridge of Mazui Mountain, 18.61°N, 109.38°E, 750 m a.s.l., 23 March 2020, *Yi-Hua Tong*, *Xue-He Ye & Ming-Zhong Huang YXH-29* (IBSC).

A key to the species of *Vaccinium* from Hainan Province is provided below.

### ﻿Key to the species of *Vaccinium* in Hainan

**Table:** 

1	Leaves serrate, chartaceous or subcoriaceous	**2**
–	Leaves entire, coriaceous	**3**
2	Secondary veins inconspicuous adaxially, 3–5 pairs; inflorescence rachis 1.4–2.5 cm long; floral bracts small, 2–3.5 mm long, caducous, lanceolate to linear, margin entire; anther tubules 1.5–2 times as long as anther thecae	***V.pseudopubicalyx***
–	Secondary veins prominent adaxially, 5–7 pairs; inflorescence rachis 4–10 cm long; floral bracts large, leaf-like, 5–20 mm long, usually persistent, ovate to oblong-ovate, margin serrate; anther tubules 2–2.5 times as long as anther thecae	***V.bracteatum***
3	Leaf apex caudate; racemes 5–9 flowered; calyx lobes lanceolate	***V.chunii***
–	Leaf apex obtuse or abruptly obtuse-acute; racemes with 10 flowers or more; calyx lobes triangular or broadly triangular	**4**
4	Leaf venation pinninerved; veins conspicuous on both sides; disk tomentose	***V.hainanense***
–	Leaf venation pinnipalmate; veins inconspicuous on both sides; disk glabrous	***V.viscifolium***

## Supplementary Material

XML Treatment for Vaccinium pseudopubicalyx

XML Treatment for Vaccinium viscifolium

## References

[B1] Dop P (1930) *Vacciniacées*. In: Lecomte H Humbert H (Eds) Flore Générale de l’Indo-Chine (Vol 3).Masson, Paris, 698–714.

[B2] Fang RC (1991) *Vaccinium*. In: Wu CY (Ed.) Flora Reipublicae Popularis Sinicae.Science Press, Beijing, Vol 57(3), 75–164.

[B3] Fang RC Stevens PF (2005) *Vaccinium*. In: Wu ZY Raven PH (Eds) Flora of China (Vol 14).Science Press, Beijing & Missouri Botanical Garden Press, St. Louis, 476–504.

[B4] Instituti Botanici Austro-Sinensis Academiae Sinicae (1974) *Vaccinium*. In: Chen HY (Ed.) Flora of Hainan (Vol.3). Science Press, Beijing, 146–148.

[B5] King G Gamble JS (1910) Materials for a Flora of the Malayan Peninsula. Journal of the Asiatic Society of Bengal 74: 1–729.

[B6] Newman M Ketphanh S Svengsuksa B Thomas P Sengdala K Lamxay V Armstrong K (2007) A checklist of the vascular plants of Lao PDR. Royal Botanic Garden, Edinburgh.

[B7] Nguyen TH (2005) 83. Ericaceae Juss. 1789. In: Ban NT (Ed.) Checklist of plant species in Vietnam (Vol.3). Agriculture Publishing House, Hanoi, 437–450.

[B8] Pham HH (1999) An illustrated flora of Vietnam. Vol. 1. Youth Publishing House, Hanoi.

[B9] POWO (2022) Plants of the World Online. https://powo.science.kew.org/taxon/urn:lsid:ipni.org:names:30000401-2 [accessed 4 Jan 2022]

[B10] Sleumer HO (1941) Vaccinioideen Studien. Botanische Jahrbücher für Systematik, Pflanzengeschichte und Pflanzengeographie 71(4): 375–510.

[B11] Sleumer HO (1961) Florae Malesianae Precursores XXVIII The genus *Vaccinium* in Malaysia. Blumea 7(1): 9–112.

[B12] Sleumer HO (1967) *Vaccinium*. In: Van Steenis CGGJ (Ed.) Flora Malesiania.Wolters-Noordhoff, Groningen, 746–878.

[B13] Stevens PF (1969) Taxonomic studies in the Ericaceae. Unpublished PhD thesis, University of Edinburgh, U.K.

[B14] Tong YH Xia NH (2015) *Vacciniumdamingshanense* sp. nov. (Ericaceae) from Guangxi, China. Nordic Journal of Botany 33(1): 74–78. 10.1111/njb.00581

[B15] Tong YH Ye XE Wu L Nguyen TTH Xia NH (2018) A new record of Ericaceae from China: *Vacciniumeberhardtii* Dop. Guihaia 38(12): 1595–1598.

[B16] Tong YH Huang YS Ye XH Cai ZY Xia NH (2020) *Vacciniumnapoense*, a new species of V.sect.Conchophyllum (Ericaceae) from Guangxi, China. Nordic Journal of Botany 2020(12): e02773. 10.1111/njb.02773

[B17] Tong YH Zhu YY Ye XH Ye XE Yang CZ Xia NH (2021a) *Vacciniumzhangzhouense*, a new species endemic to Fujian, China. Nordic Journal of Botany 39(7): e03091. 10.1111/njb.03091

[B18] Tong YH Zhao WL Wang BM Liu ED Cai J Guo YJ (2021b) *Vacciniummotuoense* (Ericaceae), a new species from Xizang, China. PhytoKeys 181: 105–111. 10.3897/phytokeys.181.71522 34611456 PMC8448724

[B19] Vander Kloet SP Dickinson TA (2009) A subgeneric classification of the genus *Vaccinium* and the metamorphosis of V.sectionBracteata Nakai: More terrestrial and less epiphytic in habit, more continental and less insular in distribution. Journal of Plant Research 122(3): 253–268. 10.1007/s10265-008-0211-7 19184674

[B20] Watthana S (2015) Ericaceae. In: Newman M Barfod A (Eds) Flora of Thailand.(Vol. 13 (part 1)). The Forest Herbarium, Department of National Parks, Wildlife and Plant Conservation, Bangkok, 101–141.

[B21] Xing FW Zhou JS Wang FG Zeng QW Yi QF Liu DM (2012) Inventory of Plant Species Diversity of Hainan. Huazhong University of Science and Technology Press, Wuhan.

[B22] Zhang RJ Ng SC Ye YS Xing FW Chen HF (2007) Newly recorded plants from Hainan Island, China (XI). Journal of Tropical and Subtropical Botany 15(3): 256–258.

